# Phenotypic and Ecological Correlates of Population Decline in the World's Anurans

**DOI:** 10.1002/ece3.73168

**Published:** 2026-03-02

**Authors:** Giovanna Sandretti‐Silva, Andreas Schwarz Meyer, Fernanda S. Caron, Raquel Divieso, Marcos R. Bornschein, Marcio R. Pie

**Affiliations:** ^1^ Institute for Biology and Environmental Sciences Carl von Ossietzky Universität Oldenburg Oldenburg Germany; ^2^ African Climate and Development Initiative University of Cape Town Cape Town Western Cape South Africa; ^3^ Departamento de Zoologia Universidade Federal do Paraná Paraná Brazil; ^4^ Department of Biodiversity and Conservation Real Jardín Botánico (CSIC) Madrid Spain; ^5^ Departamento de Ciências Biológicas e Ambientais Universidade Estadual Paulista São Vicente Brazil

**Keywords:** amphibians, body size, environmental prevalence, extinction risk, moisture, phylogeny, range size, temperature

## Abstract

Anurans are profoundly affected by the ongoing biodiversity crisis. Understanding the drivers of their population decline is key to guiding management strategies and prioritize conservation efforts. Population trends have recently become a popular indicator of extinction risk, yet comprehensive global‐scale assessments are still scarce, particularly those that account for phylogenetic nonindependence. In this study, we assess the ecological and environmental factors associated with population decline in the world's anurans. We conducted a phylogenetic generalized least squares analysis using large‐scale datasets of population trend (as indicated by their IUCN status), morphology, geographical distribution, and climate variables across 5246 globally distributed species. Specifically, we tested whether body size (BS), range size, annual mean temperature (AMT), temperature annual range (TAR), climate moisture index (CMI), latitude, and environmental prevalence (i.e., relative availability of climate conditions in the geographical space) affect population trends. A large majority of evaluated species were in decline. Range size and TAR were negatively correlated with decline, whereas latitude was positively correlated. Climatic prevalence was not correlated with decline, although declining species often showed lower prevalence values. The findings underscore the critical state of anuran populations, which may worsen in the future due to synergistic effects with climate change. Therefore, we recommend initiatives, such as the establishment of protected areas with multiple narrowly‐distributed species, and the increase of the population trend assessment coverage.

## Graphical abstract

In this study, we assess the ecological and environmental factors associated with population decline in the world's anurans, taking into account their evolutionary relationships. A large majority of evaluated species were in decline. Range size and temperature annual range were negatively correlated with decline, whereas latitude was positively correlated.

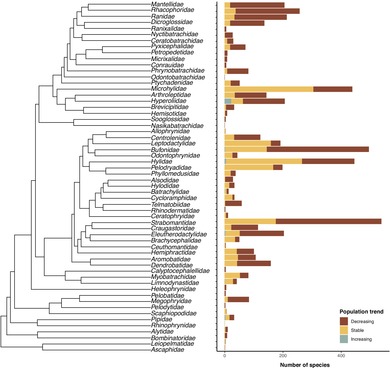

## 1 Introduction

We are experiencing a global biodiversity crisis, marked by species declines and extinction rates that are orders of magnitude higher than natural background levels (Dirzo et al. 2014; Alroy 2015; Ceballos et al. 2015; Ceballos et al. 2017; Finn et al. 2023). Central to addressing the attenuation of this crisis is the necessity of monitoring species to establish conservation priorities and allocate limited resources effectively (Caviedes‐Solis et al. 2020; Munstermann et al. 2021). Historically, the most widely employed metric for monitoring species has been the IUCN threat categories, which classify species based on criteria related to their population and geographic status, reflecting their threat levels (IUCN Standards and Petitions Committee 2024). Another metric used in IUCN assessments, though not directly applied as a criterion, is population trend (IUCN 2013). This metric assesses the trajectories' direction of populations of a given species over a short period of time around the present and reflects the threats in a more dynamic way (IUCN 2013; Ceballos et al. 2017; Finn et al. 2023). This metric is informative as it allows for the identification of species in time to establish conservation priorities and act before reaching the no‐persistence threshold of the extinction process (Gilpin and Soulé 1986; Ceballos et al. 2017; Finn et al. 2023).

Species characterized by declining populations can be associated with factors such as small distributions, limited mobility, and specialist habits. These factors influence the species' vulnerability to extrinsic threats, such as habitat modifications, human presence, invasive species, and climate change (Murray and Hose 2005; Murray et al. 2011, 2014; Munstermann et al. 2021). Understanding the factors correlated with declining populations is important to guide conservation actions and identify priority species (Cardillo et al. 2008; Murray et al. 2014; Munstermann et al. 2021).

Among vertebrates, amphibians are the most impacted group by the global biodiversity crisis, with the majority of species characterized as threatened and declining (Finn et al. 2023). Anura, the largest order of amphibians (Frost 2024), was estimated to have experienced almost 200 extinctions since 1970, with the loss of nearly 7% of species predicted to occur within the next century (Alroy 2015). For anurans, body size, habits, reproductive characteristics, geographical and altitudinal range, and environmental conditions of their distribution area have been suggested to be important factors influencing their vulnerability (Bielby et al. 2008; Cooper et al. 2008; Sodhi et al. 2008; Murray et al. 2011; Anjos et al. 2020; Guirguis et al. 2022). However, most studies on anurans (or amphibians in general) are either assessments using the IUCN threat categories or other risk assessment metrics (e.g., Hero et al. 2005; Bielby et al. 2008; Cooper et al. 2008; Whitfield et al. 2016; Anjos et al. 2020; Caviedes‐Solis et al. 2020; Cardillo 2020; Guirguis et al. 2022). In addition, either these assessments are not on a global scale (e.g., Lips et al. 2003; Murray and Hose 2005; Murray et al. 2011; Fontana et al. 2021) or are on a global scale but have a limited account of phylogenetic relatedness (e.g., Sodhi et al. 2008) or a limited number of species due to the unavailability of some of the data used in the analyses (e.g., Pincheira‐Donoso et al. 2022). Despite the importance of these studies, a global‐scale study analysis of anuran population trends considering a range of species and robust phylogenetic relatedness is still needed.

Here, we evaluate phenotypic and ecological correlates of population decline in the world's anurans. We hypothesize that large bodies and small range sizes (Sodhi et al. 2008; Murray et al. 2011; Pincheira‐Donoso et al. 2022) are the main correlates of population declines, and that low climatic prevalence (i.e., rare climate conditions, sensu Meyer and Pie 2018) are also correlated with declines because smaller areas support fewer individuals and smaller populations (Coelho et al. 2023), which are more vulnerable (MacArthur and Wilson 1967).

## 2 Materials and Methods

### 2.1 Data Collection

The dataset was organized based on the species included in the phylogeny estimated by Jetz and Pyron (2018). Population trends and threat categories of the studied species were obtained using the *rl_search* function in *rredlist 0.7.1* package (Gearty and Chamberlain 2022). Species' population trends are classified as (1) increasing, (2) stable, (3) decreasing, or (4) unknown (IUCN 2013). Given the small number of species categorized with a trend of increasing population sizes (see results), our analysis disregarded all species in that category, as well as those with unknown population trends. Geographical distributions were obtained from the IUCN Red List of Threatened Species database, version 2023‐1 (IUCN 2023). All species polygons classified as uncertain by the IUCN were excluded from the analysis. Latitudinal midpoints and range sizes were calculated using the *gCentroid* and *gArea* functions in *rgeos 0.6–4* package (Bivand and Rundel 2023).

Both climatic and ecological data were used as potential correlates of population decline. Body size (BS) was obtained from AmphiBIO (Oliveira et al. 2017) and the Amphibian traits database (Huang et al. 2023). Mean BS values of adult individuals of each species were calculated from the Amphibian traits database to obtain a single estimate from that source. The correlation between the BS values from both databases was plotted to identify potential incongruences. As a result of this methodological analysis, the estimate for *Rupirana cardosoi* from AmphiBIO was adjusted from 250 mm to 25 mm. Subsequently, we calculated the mean BS values across both databases to obtain a single body size estimate for each species. We log‐transformed BS values prior to reducing skewness in their distribution.

Annual mean temperature (AMT) and temperature annual range (TAR; defined as the difference between the maximum temperature of the warmest month and the minimum temperature of the coldest month) data across the range of each species at two resolutions (2.5 arc‐min and 10 arc‐min) were obtained from WorldClim using the *worldclim_global* function in *geodata* 0.5–9 package (Hijmans et al. 2023). Additionally, the climate moisture index (CMI) was obtained at resolutions of 30 arc‐sec from Chelsa Climate (Brun et al. 2022) and resampled to a 10 arc‐min resolution using nearest neighbor resampling in QGIS (https://qgis.org/). Mean estimates of AMT and TAR for each species were derived from the 2.5 arc‐min raster data, whereas mean CMI values were sourced from the 30 arc‐sec data. The estimates were derived by extracting data from the cells where the species were present and computing mean values across their entire geographic range. Some species lacked a minimum range size necessary for estimating AMT and TAR at the available resolution (see below).

We used phylogenetic data imputation to enhance our dataset regarding BS (*N* = 754 species), AMT and TAR (*N* = 281 species), CMI (*N* = 7 species), and population trends (*N* = 1401 species), given the improved statistical performance of downstream analyses (e.g., Penone et al. 2014; May et al. 2023). For this purpose, we used the consensus phylogeny from Jetz and Pyron (2018), which was pruned to contain only the species considered in the study. The *impute* function in *funspace 0.2.1* package (Carmona et al. 2024) was employed. To assess the reliability of the imputed values, we performed a leave‐one‐out cross‐validation procedure. This involved systematically removing known values for each species and re‐imputing them to compare observations with predictions. For continuous variables, we evaluated performance using the predictive *R* ^2^, while the accuracy of population trend predictions was assessed using a confusion matrix.

The environmental prevalence of temperature and moisture variables was calculated by assessing the geographical extent of different environmental conditions according to Meyer and Pie (2018). We used rasters at a resolution of 10 min in a cylindrical equal‐area projection and excluded Antarctica from the analysis. We considered 60 equally spaced intervals between −27°C and 31°C (MAT), and −312 to 390 kg m^−2^ month^−1^ (CMI). The lowest temperature and highest moisture environmental intervals were lumped together, given their rare prevalence. The prevalence of the mean climatic estimates for each species was obtained by comparing them with the climatic prevalence of the closest value corresponding to their climatic range.

### 2.2 Data Analysis

First, we assessed the association between IUCN conservation status and population trends using a Phylogenetic Logistic Regression to account for phylogenetic non‐independence. These models were fitted using the *phyloglm* function from the phylolm package v2.6‐5 (Ho and Ane 2014), employing the Maximum Clade Credibility (MCC) tree. This analysis was performed for both the original and imputed datasets to evaluate potential discrepancies between formal threat categories and observed demographic trends.

Subsequently, to account for the binary nature of population trends (Ives and Garland 2010), we fitted MCMC Generalized Linear Mixed Models using the *MCMCglmm* function of the *MCMCglmm* package v2.36 (Hadfield 2010) to assess the relationship between species population trends and morphological, ecological, and climatic variables. We adopted a categorical family for the response variable and included the phylogeny as a random effect by calculating the inverse of the phylogenetic covariance matrix using the *inverseA* function of the *MCMCglmm* package v2.36 (Hadfield 2010). Our full model contained BS, range size, absolute latitude midpoint, AMT, TAR, CMI, prevalence of AMT, and prevalence of CMI as predictors. To fit this model and its subsets, BS and range size were log transformed, whereas AMT, TAR, CMI, prevalence of AMT, prevalence of CMI, and absolute latitude were scaled.

To perform model selection, we compared all 256 possible model combinations for both the original and imputed datasets using the Maximum Clade Credibility (MCC) tree. Selection was based on the Deviance Information Criterion (DIC), choosing the model with ΔDIC < 2. The final selected models were then validated across a posterior distribution of 100 random posterior topologies from Jetz and Pyron (2018) to account for phylogenetic uncertainty. For all MCMCglmm iterations, we used a total of 1,000,000 iterations, with a burn‐in of 100,000 and a thinning interval of 500. We used weakly informative priors with a parameter‐expansion approach for the phylogenetic component and a fixed residual variance, following standard recommendations for categorical response variables in MCMCglmm. Model convergence was confirmed through the evaluation of trace plots and effective sample sizes for all parameters. All analyses were conducted using R version 4.4.0 (R Core Team 2024), and the complete data and code for these procedures are available in the GitHub repository https://github.com/fernandacaron/anuran_population_decline.

## 3 Results

**FIGURE 1:**
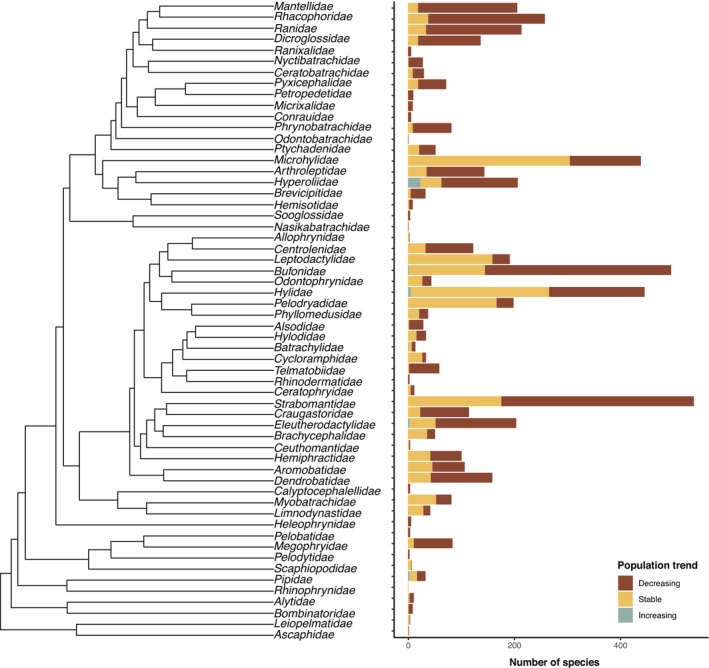
Distribution of the world's anuran species across population trends by family (*N* = 5246).

Among the 3845 species with available population trend data, 2335 (61%) were declining, 1489 (38%) were stable, and 21 (< 0.1%) were increasing. Among the 36 families with more than 10 represented species, the average proportion of declining species ranged from 15.9% (Leptodactylidae) to 96.3% (Telmatobiidae), with most of them (66.7%) exhibiting over half of their species in decline (61.9%; Figure 1). We found a significant association between population trends and IUCN threat categories (imputed dataset: *p* < 0.001; non‐imputed dataset: *p* < 0.001). In the imputed model (*N* = 5206), all categories showed significantly higher stability compared to the Critically Endangered (CE) intercept (*p* < 0.001), except for Endangered (EN) species (*p* = 0.261). Despite this general alignment, a notable discrepancy was observed: 31% of the species exhibiting decreasing population trends are currently classified as Least Concern (LC). Conversely, 91% of species with stable trends belong to non‐threatened categories (LC and NT). Among Data Deficient (DD) species, 74% exhibit decreasing population trends.

**FIGURE 2:**
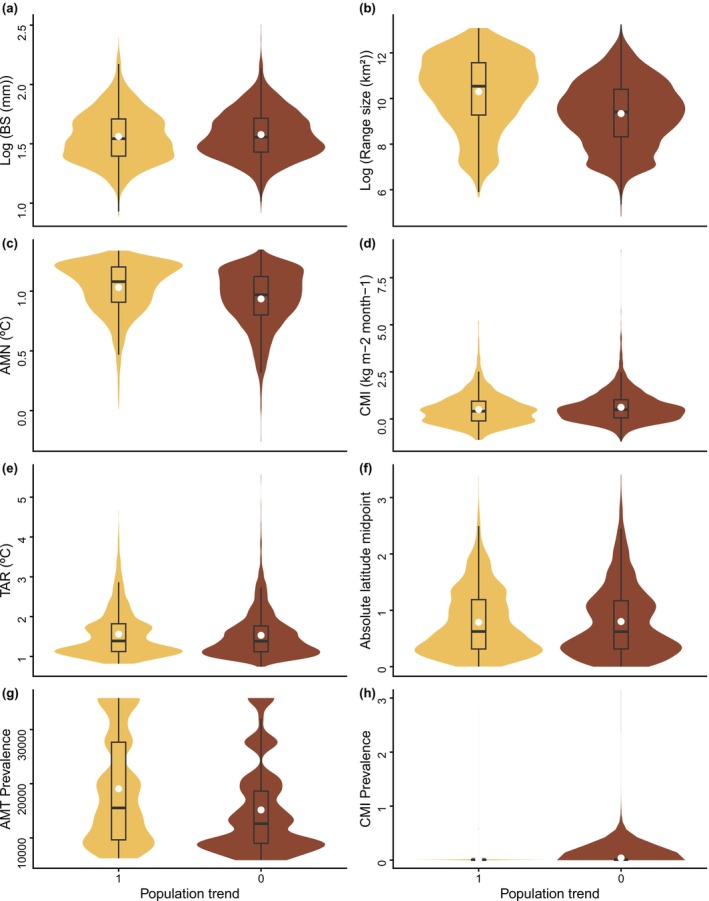
Relationship between population trends of the world's anurans and the potential factors associated with decline: (a) body size, (b) range size, (c) temperature, (d) moisture, (e) temperature range, (f) absolute latitude, (g) temperature prevalence, and (h) moisture prevalence. AMT, annual mean temperature; BS, body size; CMI, climate moisture index; TAR, temperature annual range.

The performance of our phylogenetic imputation showed high reliability for TAR (*R* ^2^ = 0.853), AMT (*R* ^2^ = 0.632), and BS (*R* ^2^ = 0.575), with a moderate performance for moisture (*R* ^2^ = 0.486). The accuracy of population trend predictions yielded an overall accuracy of 79.3% and a Kappa statistic of 0.561. This suggests that our phylogenetic imputation approach provides reliable estimates for both morphological and environmental traits, as well as for species without current population status information. Accordingly, the imputed data indicated that, of all 1401 species with unknown population trends, the majority are likely to be declining (*N* = 908), followed by being stable (*N* = 474), and increasing (*N* = 19) (Figure 2).

### 3.1 Correlates of Population Decline

The model selection based on DIC identified different best‐supported models for the original and imputed datasets. For the imputed dataset, a single best‐supported model was identified, which included range size, AMT, TAR, CMI, prevalence of AMT, prevalence of CMI, and latitude as predictors. For the non‐imputed dataset, multiple models showed comparable support (ΔDIC < 2). In this case, we selected the most biologically informative model among the top candidates, specifically the one that included key climatic predictors, which presented 95% credible intervals not overlapping zero. Therefore, the chosen best‐fit model included range size, AMT, CMI, prevalence of AMT, prevalence of CMI, and latitude as predictors, differing from the imputed dataset only in the absence of TAR.

**FIGURE 3:**
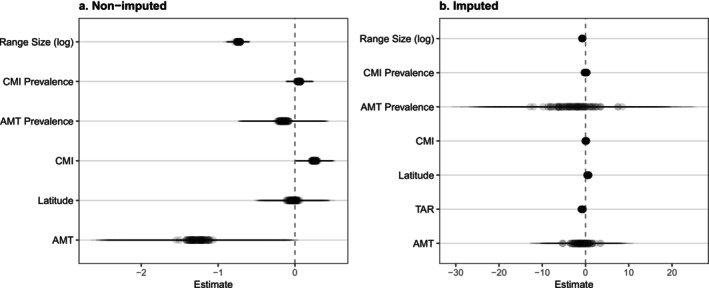
Predictors of population decline in amphibians. Regression estimates from MCMCglmm for (a) non‐imputed and (b) imputed datasets. Points indicate posterior means, and horizontal bars represent 95% credible intervals. Overlapping points and bars represent statistics calculated across 100 alternative topologies. Predictors are considered significant when intervals do not overlap the dashed zero line. AMT, annual mean temperature; CMI, climate moisture index; TAR, temperature annual range.

For the non‐imputed dataset, population decline was significantly associated with range size, AMT, and CMI (Figure 3; Table S1). Species with smaller range sizes showed a higher probability of population decline (posterior mean (PM) across topologies = −0.735; 95% CI = [−0.870, −0.606]). Similarly, higher AMT was correlated with decline (PM = –1.269; 95% CI = [−2.393, −0.151]), while higher CMI had a buffering effect on population decline (PM = 0.252; 95% CI = [0.028, 0.476]). Other predictors, such as latitude and climatic prevalence, did not show significant effects, as their 95% credible intervals overlapped zero (Figure 2). However, given that the imputation process demonstrated good performance, we focus primarily on the results from the imputed dataset, which provides a more comprehensive assessment by accounting for missing trait data. Thus, the imputed dataset results were characterized by high uncertainty in climatic predictors (Figure 3; Table S1). Although range size remained a consistent and significant predictor of decline (PM = –0.740; 95% CI = [−0.855, −0.632]), the effects of AMT and its prevalence showed extremely wide credible intervals, overlapping zero. Notably, TAR and absolute latitude emerged as significant predictors in the imputed model, in which species in regions with higher temperature seasonality (PM = –0.781; 95% CI = [−1.239, −0.327]) and those located at lower latitudes (PM = 0.534; 95% CI = [0.094, 0.984]) were more likely to exhibit decreasing population trends. The phylogenetic signal was substantial in both approaches, with the posterior mean of the phylogenetic variance component being slightly higher in the imputed dataset (12.82) compared to the non‐imputed (10.13).

## 4 Discussion

Our study employed a robust phylogenetic approach to identify key phenotypic and ecological correlates of population decline in anurans at a global scale. Our analysis revealed that range size and TAR are negative correlates of species decline, and latitude is a positive correlate. Contrary to our hypothesis, the climatic prevalences were found not to be significant correlates. However, it is interesting to note that declining species exhibited lower average prevalence values compared to stable species (Figure 2), suggesting a potential influence of these factors.

Positive relationships between BS and vulnerability or decline have been found by some authors (e.g., Lips et al. 2003; Murray and Hose 2005; Sodhi et al. 2008; Guirguis et al. 2022; Pincheira‐Donoso et al. 2022), but they are not universally observed (Pincheira‐Donoso and Hodgson 2018; Anjos et al. 2020; Cardillo 2020; Fontana et al. 2021). The most important correlate found in our study was the range size, which agrees with most previous findings (e.g., Hero et al. 2005; Murray and Hose 2005; Bielby et al. 2008). Small distributions are associated with an increased exposure to external stochastic forces, inbreeding, and habitat specificity, and decreased population size, density and dispersal, leading to low values of reproductive success, survival and colonization (Cooper et al. 2008; Courchamp et al. 2008; McCauley et al. 2014; Whitfield et al. 2016). Additionally, the distribution maps from the IUCN, utilized for assessing range size, represent the current known distribution of species within their native range (IUCN 2013). Therefore, significant declines that lead to smaller current ranges, regardless of the species' original range sizes, likely correlate with population declines.

Climate variables have frequently been associated with amphibian vulnerability to extinction (Bielby et al. 2008; Cooper et al. 2008; Sodhi et al. 2008; Pincheira‐Donoso et al. 2022). For example, lower temperatures and more humid conditions are associated with the prevalence and intensity of *B. dendrobatidis* infection in anurans (Riley et al. 2013; Kolby et al. 2015), which may explain the observed relationship for these variables. The TAR as a negative correlate of decline can be considered a case of specialist vulnerability (Clavel et al. 2011) of species with narrow thermal breadths. Moreover, species with narrow thermal breadths may more easily suffer from increased *B. dendrobatidis* infection during unusual temperature conditions (Cohen et al. 2019).

The population declines in most studied species, even those not currently considered threatened, underscore the importance of conservation actions to prevent extinctions that are already underway and highlight the value of population trends for a more realistic assessment of species conservation status (Ceballos et al. 2017; Finn et al. 2023). Reductions in anuran distribution areas are tightly linked to human activities, including habitat loss and fragmentation driven by agricultural expansion, deforestation, urbanization and infrastructure development, as well as pollution, overexploitation, and the human‐mediated spread of pathogens (e.g., Sodhi et al. 2008; Hof et al. 2011). These processes erode both the quantity and quality of suitable habitats, isolate subpopulations, and limit opportunities for recolonization, thereby magnifying the vulnerability of species with already small ranges (Fahrig 2003). The identification of range size as the primary negative correlate of population decline therefore likely reflects the cumulative impact of anthropogenic habitat modification on narrowly distributed species and supports the rationale for establishing protected areas that encompass multiple range‐restricted taxa, thus improving the allocation of limited conservation resources (Cooper et al. 2008; Bornschein et al. 2019). In addition, the significance of climatic variables as correlates raises concerns regarding ongoing human‐driven climate change, which will disproportionately affect species in the future, particularly when combined with other threats such as chytridiomycosis and habitat fragmentation (Dirzo et al. 2014; Munstermann et al. 2021).

The environmental prevalence of AMT and CMI did not correlate with the observed decline in anuran populations, despite the decreased prevalence observed for declining species. These findings contradict our initial hypothesis regarding prevalence and suggest that other factors, such as range size, have a greater influence on determining population decline. However, it is important to consider that environmental prevalence also changes with climate change, altering the distribution of climatic conditions (Ackerly et al. 2010). Therefore, it would be relevant for future studies to investigate how the prevalence range and speed of variation over time impact population declines, particularly considering that species' evolution occurs at a much slower rate than the changes (Meyer and Pie 2022).

The abundance of species with unknown population trends underscores the urgency of improving the coverage and quality of population trend assessments. However, our reliance on the IUCN “Population Trend” field entails important limitations: these trends are not derived from standardized global monitoring, but often from expert judgment, literature summaries, or indirect inference, and they are updated irregularly. As a consequence, population trend categories are not strictly comparable among regions or taxa, particularly given strong heterogeneity in survey effort (e.g., more systematic monitoring in some temperate regions versus anecdotal or museum‐based records in parts of the tropics). Moreover, by excluding species classified as having unknown or increasing trends, our dataset is likely biased towards relatively well‐studied species and regions, potentially underrepresenting some of the most at‐risk clades and areas. These caveats do not invalidate our conclusions, but they indicate that our results should be interpreted as applying primarily to the subset of anuran species for which sufficient information is available. It is highly likely that many species currently classified as having unknown population trends are in fact declining, as they are concentrated in tropical areas that have large numbers of declining populations (Finn et al. 2023). Thus, many of these species may be heading towards extinction and may not receive attention from conservation actions because they are not yet assessed as threatened (Bland et al. 2014). An additional caveat of our analyses is that several predictors represent biologically and environmentally linked gradients and are therefore not fully independent. For instance, body size, climatic variables, and geographic position can covary across species due to shared macroecological and evolutionary processes, meaning that some predictors may act as partial proxies for broader underlying gradients. Although model selection reduces the likelihood that highly redundant variables are retained simultaneously, the remaining coefficients should be interpreted as conditional effects rather than isolated causal drivers. Consequently, the detected associations likely reflect the combined influence of correlated life‐history, climatic, and biogeographic factors, and caution is warranted when attributing mechanistic primacy to any single predictor.

In this study, we highlight the crisis that anuran populations are facing. We showed that decreased range size, TAR, and latitude are correlated with population declines, offering insights for prioritizing conservation actions, such as the creation of protected areas that encompass multiple narrowly distributed species. Although climate prevalences were not a significant correlate of population decline, the observed relationship between declining species and lower climatic prevalences raises concerns about the future impacts of climate change on anurans. We argue that expanding population trend assessments and investigating the interaction between environmental factors and population declines are crucial steps to develop and implement effective urgent actions for biodiversity conservation.

## Author Contributions

**Giovanna Sandretti‐Silva:** data curation (lead), formal analysis (equal), methodology (equal), visualization (lead), writing – original draft (equal), writing – review and editing (equal). **Andreas Schwarz Meyer:** formal analysis (equal), methodology (equal), writing – review and editing (equal). **Fernanda S. Caron:** data curation (supporting), formal analysis (equal), methodology (equal), visualization (supporting), writing – original draft (supporting), writing – review and editing (equal). **Raquel Divieso:** data curation (supporting), writing – review and editing (equal). **Marcos R. Bornschein:** writing – review and editing (equal). **Marcio R. Pie:** conceptualization (equal), formal analysis (equal), methodology (equal), supervision (equal), writing – original draft (equal), writing – review and editing (equal).

## Funding

This work was supported by Fundação de Amparo à Pesquisa do Estado de São Paulo (2022/04847‐7, 2023/09718‐3) and Coordinação de Aperfeiçoamento de Pessoal de Nível Superior (88887.923452/2023‐00). M.R.P. was funded by a Productivity Fellowship from the Conselho Nacional de Desenvolvimento Científico e Tecnológico (303491/2024‐8).

## Conflicts of Interest

The authors declare no conflicts of interest.

## Supporting information


**Data S1:** ece373168‐sup‐0001‐supinfo.docx.

## Data Availability

All data and R scripts required to reproduce the analyses are available in the GitHub repository https://github.com/fernandacaron/anuran_population_decline.

## References

[ece373168-bib-0001] Ackerly, D. D. , S. R. Loarie , W. K. Cornwell , et al. 2010. “The Geography of Climate Change: Implications for Conservation Biogeography.” Diversity and Distributions 16: 476–487. 10.1111/j.1472-4642.2010.00654.x.

[ece373168-bib-0002] Alroy, J. 2015. “Current Extinction Rates of Reptiles and Amphibians.” PNAS 112: 13003–13008. 10.1073/pnas.1508681112. 26438855 PMC4620882

[ece373168-bib-0003] Anjos, A. G. , R. N. Costa , D. Brito , and M. Solé . 2020. “Is There an Association Between the Ecological Characteristics of Anurans From the Brazilian Atlantic Forest and Their Extinction Risk?” Ethology Ecology & Evolution 32: 336–350. 10.1080/03949370.2020.171181.

[ece373168-bib-0004] Bielby, J. , N. Cooper , A. A. Cunningham , T. W. J. Garner , and A. Puvis . 2008. “Predicting Susceptibility to Future Declines in the World's Frogs.” Conservation Letters 1: 82–90. 10.1111/j.1755-263X.2008.00015.x.

[ece373168-bib-0005] Bivand, R. , and C. Rundel . 2023. “rgeos: Interface to Geometry Engine—Open Source (‘GEOS’).” R Package, Version 0.6‐4. https://CRAN.R‐project.org/package=rgeos.

[ece373168-bib-0006] Bland, M. L. , B. Collen , C. D. L. Orme , and J. Bielby . 2014. “Predicting the Conservation Status of Data‐Deficient Species.” Conservation Biology 29: 250–259. 10.1111/cobi.12372. 25124400

[ece373168-bib-0007] Bornschein, M. R. , M. R. Pie , and L. Teixeira . 2019. “Conservation Status of *Brachycephalus* Toadlets (Anura: Brachycephalidae) From the Brazilian Atlantic Rainforest.” Diversity 11: 150. 10.3390/d11090150.

[ece373168-bib-0008] Brun, P. , N. E. Zimmermann , C. Hari , L. Pellissier , and D. N. Karger . 2022. “Global Climate‐Related Predictors at Kilometer Resolution for the Past and Future.” Earth System Science Data 14: 5573–5603. 10.5194/essd-14-5573-2022.

[ece373168-bib-0009] Cardillo, M. 2020. “Clarifying the Relationship Between Body Size and Extinction Risk in Amphibians by Complete Mapping of Model Space.” Proceedings Biological Sciences 288: 20203011. 10.1098/rspb.2020.3011. PMC7893221 33529561

[ece373168-bib-0010] Cardillo, M. , G. M. Mace , J. L. Gittleman , K. E. Jones , J. Bielby , and A. Purvis . 2008. “The Predictability of Extinction: Biological and External Correlates of Decline in Mammals.” Proceedings of the Royal Society B 275: 1441–1448. 10.1098/rspb.2008.0179. 18367443 PMC2602711

[ece373168-bib-0011] Carmona, C. P. , N. Pavanetto , and G. Puglielli . 2024. “funspace: Creating and Representing Functional Trait Spaces.” R Package, Version 0.2.1. https://cran.r‐project.org/package=funspace.

[ece373168-bib-0012] Caviedes‐Solis, I. , N. Kim , and A. D. Leaché . 2020. “Species IUCN Threat Status Level Increases With Elevation: A Phylogenetic Approach for Neotropical Tree Frog Conservation.” Biodiversity and Conservation 29: 2515–2537. 10.1007/s10531-020-01986-8.

[ece373168-bib-0013] Ceballos, G. , P. R. Ehrilich , A. D. Barnosky , A. García , R. M. Pringle , and T. D. Palmer . 2015. “Accelerated Modern Human–Induced Species Losses: Entering the Sixth Mass Extinction.” Science Advances 1: e1400253. 10.1126/sciadv.1400253. 26601195 PMC4640606

[ece373168-bib-0014] Ceballos, G. , P. R. Ehrilich , and R. Dirzo . 2017. “Biological Annihilation via the Ongoing Sixth Mass Extinction Signaled by Vertebrate Population Losses and Declines.” PNAS 114: E6089–E6096. 10.1073/pnas.1704949114. 28696295 PMC5544311

[ece373168-bib-0015] Clavel, J. , R. Julliard , and V. Devictor . 2011. “Worldwide Decline of Specialist Species: Toward a Global Functional Homogenization?” Frontiers in Ecology and the Environment 9: 222–228. 10.1890/080216.

[ece373168-bib-0016] Coelho, M. T. P. , E. Barreto , T. F. Rangel , et al. 2023. “The Geography of Climate and the Global Patterns of Species Diversity.” Nature 622: 537–544. 10.1038/s41586-023-06577-5. 37758942 PMC10584679

[ece373168-bib-0017] Cohen, J. M. , T. A. McMahon , C. Ramsay , et al. 2019. “Impacts of Thermal Mismatches on Chytrid Fungus *Batrachochytrium dendrobatidis* Prevalence Are Moderated by Life Stage, Body Size, Elevation and Latitude.” Ecology Letters 22: 817–825. 10.1111/ele.13239. 30816626

[ece373168-bib-0018] Cooper, N. , J. Bielby , G. A. Thomas , and A. Purvis . 2008. “Macroecology and Extinction Risk Correlates of Frogs.” Global Ecology and Biogeography 17: 211–221. 10.1111/j.1466-8238.2007.00355.x.

[ece373168-bib-0020] Courchamp, F. , L. Berec , and J. Gascoigne . 2008. Allee Effects in Ecology and Conservation. Sinauer Associates Inc.

[ece373168-bib-0021] Dirzo, R. , H. S. Young , M. Galetti , G. Ceballos , N. J. Isaac , and B. Collen . 2014. “Defaunation in the Anthropocene.” Science 345: 401–406. 10.1126/science.1251817. 25061202

[ece373168-bib-0022] Fahrig, L. 2003. “Effects of Habitat Fragmentation on Biodiversity.” Annual Review of Ecology, Evolution, and Systematics 34: 487–515. 10.1146/annurev.ecolsys.34.011802.132419.

[ece373168-bib-0023] Finn, C. , F. Grattalora , and D. Pincheira‐Donosom . 2023. “More Losers Than Winners: Investigating Anthropocene Defaunation Through the Diversity of Population Trends.” Biological Reviews 98: 1732–1748. 10.1111/brv.12974. 37189305

[ece373168-bib-0024] Fontana, R. B. , R. Furtado , N. Zanella , V. J. Debastiani , and S. M. Hartzm . 2021. “Linking Ecological Traits to Extinction Risk: Analysis of a Neotropical Anuran Database.” Biological Conservation 264: 109390. 10.1016/j.biocon.2021.109390.

[ece373168-bib-0025] Frost, D. R. 2024. Amphibian Species of the World: An Online Reference. Version 6.2. American Museum of Natural History.

[ece373168-bib-0026] Gearty, W. , and S. Chamberlain . 2022. “Rredlist: IUCN' Red List Client.” R Package, Version 0.7.1. https://cran.r‐project.org/package=rredlist.

[ece373168-bib-0027] Gilpin, M. E. , and M. E. Soulé . 1986. “Minimum Viable Populations: Processes of Species Extinction.” In Conservation Biology: The Science of Scarcity and Diversity, edited by M. E. Soulé , 19–34. Sinauer Associates Inc.

[ece373168-bib-0028] Guirguis, J. , L. E. B. Goodyear , C. Finn , J. V. Johnson , and D. Pincheira‐Donoso . 2022. “Risk of Extinction Increases Towards Higher Elevations Acrossthe World's Amphibians.” Global Ecology and Biogeography 32: 1954–1963. 10.1111/geb.13746.

[ece373168-bib-0029] Hadfield, J. D. 2010. “MCMC Methods for Multi‐Response Generalized Linear Mixed Models: The MCMCglmm R Package.” Journal of Statistical Software 33: 1–22. 10.18637/jss.v033.i02. 20808728 PMC2929880

[ece373168-bib-0030] Hero, J. M. , S. E. Williams , and W. E. Magnusson . 2005. “Ecological Traits of Declining Amphibians in Upland Areas of Eastern Australia.” Journal of Zoology 267: 221–232. 10.1017/S0952836905007296.

[ece373168-bib-0031] Hijmans, R. J. , M. Barbosa , A. Ghosh , and A. Mandel . 2023. “Geodata: Download Geographic Data.” R Package, Version 0.5–9. https://CRAN.R‐project.org/package=geodata.

[ece373168-bib-0032] Ho, L. S. T. , and C. Ane . 2014. “A Linear‐Time Algorithm for Gaussian and Non‐Gaussian Trait Evolution Models.” Systematic Biology 63: 397–408. 10.1093/sysbio/syu005. 24500037

[ece373168-bib-0033] Hof, C. , M. B. Araújo , W. Jetz , and C. Rahbeck . 2011. “Additive Threats From Pathogens, Climate and Land‐Use Change for Global Amphibian Diversity.” Nature 480: 516–519. 10.1038/nature10650. 22089134

[ece373168-bib-0034] Huang, N. , X. Sun , Y. Song , Z. Yuan , and W. Zhou . 2023. “Amphibian Traits Database: A Global Database on Morphological Traits of Amphibians.” Global Ecology and Biogeography 32: 633–641. 10.1111/geb.13656.

[ece373168-bib-0035] IUCN . 2023. “The IUCN Red List of Threatened Species.” Version 2023‐1. Accessed January 23, 2024. https://www.iucnredlist.org.

[ece373168-bib-0036] IUCN (International Union for Conservation of Nature) . 2013. Documentation Standards and Consistency Checks for IUCN Red List Assessments and Species Accounts. Version 2. IUCN Red List Committee and IUCN SSC Steering Committee.

[ece373168-bib-0037] IUCN Standards and Petitions Committee . 2024. Guidelines for Using the IUCN Red List Categories and Criteria. Version 16. Standards and Petitions Committee.

[ece373168-bib-0038] Ives, A. R. , and T. Garland . 2010. “Phylogenetic Logistic Regression for Binary Dependent Variables.” Systematic Biology 59: 9–26. 10.1093/sysbio/syp074. 20525617

[ece373168-bib-0039] Jetz, W. , and R. A. Pyron . 2018. “The Interplay of Past Diversification and Evolutionary Isolation With Present Imperilment Across the Amphibian Tree of Life.” Nature Ecology & Evolution 2: 850–858. 10.1038/s41559-018-0515-5. 29581588

[ece373168-bib-0040] Kolby, J. E. , S. D. Ramirez , L. Berder , K. L. Richards‐Hrdlicka , M. Jocque , and L. F. Skerratt . 2015. “Terrestrial Dispersal and Potential Environmental Transmission of the Amphibian Chytrid Fungus (*Batrachochytrium dendrobatidis*).” PLoS One 10: e0125386. 10.1371/journal.pone.0125386. 25927835 PMC4415912

[ece373168-bib-0041] Lips, K. R. , J. D. Reeve , and L. R. Witters . 2003. “Ecological Traits Predicting Amphibian Population Declines in Central America.” Conservation Biology 17: 1078–1088. 10.1046/j.1523-1739.2003.01623.x.

[ece373168-bib-0058] MacArthur, R. H. , and E. O. Wilson . 1967. The Theory of Island Biogeography. Princeton University Press.

[ece373168-bib-0042] May, J. A. , Z. Feng , and S. J. Adamowicz . 2023. “A Real Data‐Driven Simulation Strategy to Select an Imputation Method for Mixed‐Type Trait Data.” PLoS Computational Biology 19: e1010154. 10.1371/journal.pcbi.1010154. 36947561 PMC10069776

[ece373168-bib-0043] McCauley, S. J. , C. J. Davis , E. E. Werner , and M. S. Robeson II . 2014. “Dispersal, Niche Breadth and Population Extinction: Colonization Ratios Predict Range Size in North American Dragonflies.” Journal of Animal Ecology 83: 858–865. 10.1111/1365-2656.12173. 24237364

[ece373168-bib-0044] Meyer, A. L. S. , and M. R. Pie . 2018. “Environmental Prevalence and the Distribution of Species Richness Across Climatic Niche Space.” Journal of Biogeography 45: 2348–2360. 10.1111/jbi.13419.

[ece373168-bib-0045] Meyer, A. L. S. , and M. R. Pie . 2022. “Climate Change Estimates Surpass Rates of Climatic Niche Evolution in Primates.” International Journal of Primatology 43: 40–56. 10.1007/s10764-021-00253-z.

[ece373168-bib-0046] Munstermann, M. J. , N. A. Heim , D. J. McCauley , et al. 2021. “A Global Ecological Signal of Extinction Risk in Terrestrial Vertebrates.” Conservation Biology 36: e13852. 10.1111/cobi.13852. 34668599 PMC9299904

[ece373168-bib-0047] Murray, B. R. , and G. C. Hose . 2005. “Life‐History and Ecological Correlates of Decline and Extinction in the Endemic Australian Frog Fauna.” Austral Ecology 30: 564–571. 10.1111/j.1442-9993.2005.01471.x.

[ece373168-bib-0048] Murray, K. A. , L. D. V. Arregoitia , A. Davidson , M. Di Marcos , and M. M. I. Donzo . 2014. “Threat to the Point: Improving the Value of Comparative Extinction Risk Analysis for Conservation Action.” Global Change Biology 20: 483–494. 10.1111/gcb.123667. 23966334

[ece373168-bib-0049] Murray, K. A. , D. Rosauer , H. McCallum , and L. F. Skerratt . 2011. “Integrating Species Traits With Extrinsic Threats: Closing the Gap Between Predicting and Preventing Species Declines.” Proceedings of the Royal Society B 278: 1515–1523. 10.1098/rspb.2010.1872. 20980304 PMC3081746

[ece373168-bib-0050] Oliveira, B. F. , V. A. São‐Pedro , G. Santos‐Barrera , C. Penone , and G. C. Costa . 2017. “AmphiBIO, a Global Database for Amphibian Ecological Traits.” Scientific Data 4: 170123. 10.1038/sdata.2017.123. 28872632 PMC5584397

[ece373168-bib-0051] Penone, C. , A. D. Davidson , K. T. Shoemaker , et al. 2014. “Imputation of Missing Data in Life‐History Trait Datasets: Which Approach Performs the Best?” Methods in Ecology and Evolution 5: 961–970. 10.1111/2041-210X.12232.

[ece373168-bib-0052] Pincheira‐Donoso, D. , L. P. Harvey , J. V. Johnson , et al. 2022. “Genome Size Does Not Influence Extinction Risk in the World's Amphibians.” Functional Ecology 37: 190–200. 10.1111/1365-2435.14247.

[ece373168-bib-0053] Pincheira‐Donoso, D. , and D. J. Hodgson . 2018. “No Evidence That Extinction Risk Increases in the Largest and Smallest Vertebrates.” Proceedings of the National Academy of Sciences 115: 5845–E5846. 10.1073/pnas.1804633115. PMC6042110 29899152

[ece373168-bib-0054] R Core Team . 2024. R: A Language and Environment for Statistical Computing. R Foundation for Statistical Computing. https://www.R‐project.org/.

[ece373168-bib-0055] Riley, K. , O. F. Berry , and J. D. Roberts . 2013. “Do Global Models Predicting Environmental Suitability for the Amphibian Fungus, *Batrachochytrium dendrobatidis*, Have Local Value to Conservation Managers?” Journal of Applied Ecology 50: 713–720. 10.1111/1365-2664.12091.

[ece373168-bib-0056] Sodhi, N. S. , D. Bickford , A. C. Diesmos , et al. 2008. “Measuring the Meltdown: Drivers of Global Amphibian Extinction and Decline.” PLoS One 3: e1636. 10.1371/journal.pone.0001636. 18286193 PMC2238793

[ece373168-bib-0057] Whitfield, S. M. , K. R. Lips , and M. A. Donnelly . 2016. “Amphibian Decline and Conservation in Central America.” Copeia 104: 351–379.

