# Supplementary material for: Phenotypic and Ecological Correlates of Population Decline in the World's Anurans
Source: Ecol Evol. 2026 Mar 2;16(3):e73168. doi: 10.1002/ece3.73168 (PMC12951123; doi:10.1002/ece3.73168)
Supplement: Supplementary file 1 — Data S1: ece373168‐sup‐0001‐supinfo.docx. [file ECE3-16-e73168-s001.docx]

**SUPPLEMENTARY MATERIAL**

**Table S1.** Results of MCMCglmm for amphibian population decline. The table presents the posterior mean, 95% credible intervals (Lower and Upper CI), and pMCMC values for both the Non-imputed and Imputed datasets. Significant predictors (pMCMC < 0.05) are highlighted in bold. AMT = Annual Mean Temperature; CMI = Climate Moisture Index; TAR = Temperature Annual Range.

| **Statistic** | **Non-imputed dataset** | | | | **Imputed dataset** | | | |
| --- | --- | --- | --- | --- | --- | --- | --- | --- |
|  | **Posterior mean** | **Lower CI** | **Upper CI** | **pMCMC** | **Posterior mean** | **Lower CI** | **Upper CI** | **pMCMC** |
| Intercept | 9.722 | 6.983 | 12.550 | **0.000** | 12.851 | 1.445 | 24.289 | **0.027** |
| Range size (log) | -0.735 | -0.870 | -0.607 | **0.000** | -0.740 | -0.855 | -0.632 | **0.000** |
| AMT | -1.270 | -2.393 | -0.151 | **0.026** | -0.872 | -9.074 | 7.270 | 0.835 |
| CMI | 0.252 | 0.028 | 0.477 | **0.027** | 0.077 | -0.215 | 0.368 | 0.603 |
| TAR | - | - | - | - | -0.781 | -1.240 | -0.328 | **0.001** |
| AMT Prevalence | -0.150 | -0.666 | 0.366 | 0.568 | -2.526 | -21.493 | 16.585 | 0.792 |
| CMI Prevalence | 0.053 | -0.100 | 0.218 | 0.517 | 0.032 | -0.706 | 0.767 | 0.934 |
| Absolute latitude | -0.023 | -0.434 | 0.389 | 0.910 | 0.534 | 0.094 | 0.985 | **0.017** |
